# Balance of *XYL1* and *XYL2* expression in different yeast chassis for improved xylose fermentation

**DOI:** 10.3389/fmicb.2012.00355

**Published:** 2012-10-05

**Authors:** Jian Zha, Meng-Long Hu, Ming-Hua Shen, Bing-Zhi Li, Jing-Yu Wang, Ying-Jin Yuan

**Affiliations:** ^1^Key Laboratory of Systems Bioengineering (Tianjin University), Ministry of Education, Department of Pharmaceutical Engineering, School of Chemical Engineering and Technology, Tianjin UniversityTianjin, P. R. China; ^2^Department of Biochemical Engineering, School of Chemical Engineering and Technology, Tianjin UniversityTianjin, P. R. China

**Keywords:** pathway balance, chassis, xylose reductase, xylitol dehydrogenase, xylose, ethanol

## Abstract

Reducing xylitol formation is necessary in engineering xylose utilization in recombinant *Saccharomyces cerevisiae* for ethanol production through xylose reductase/xylitol dehydrogenase pathway. To balance the expression of *XYL1* and mutant *XYL2* encoding xylose reductase (XR) and NADP^+^-dependent xylitol dehydrogenase (XDH), respectively, we utilized a strategy combining chassis selection and direct fine-tuning of *XYL1* and *XYL2* expression in this study. A *XYL1* gene under the control of various promoters of *ADH1*, truncated *ADH1* and *PGK1*, and a mutated *XYL2* with different copy numbers were constructed into different xylose-utilizing modules, which were then expressed in two yeast chassises W303a and L2612. The strategy enabled an improved L2612-derived recombinant strain with *XYL1* controlled by promoter *PGK1* and with two copies of *XYL2*. The strain exhibited a 21.3% lower xylitol yield and a 40.0% higher ethanol yield. The results demonstrate the feasibility of the combinatorial strategy for construction of an efficient xylose-fermenting *S. cerevisiae*.

## Introduction

Efficient utilization of xylose is important for economic fermentation of biomass to fuels and chemicals (Jeffries, 2006; Chu and Lee, 2007; Stephanopoulos, 2007; Fischer et al., 2008). *Saccharomyces cerevisiae* (*S. cerevisiae*) is commonly used for industrial ethanol fermentation. However, because *S. cerevisiae* is not able to assimilate xylose naturally, engineering *S. cerevisiae* for efficient xylose utilization by introducing a xylose pathway from xylose-fermenting yeasts such as *Scheffersomyces stipitis* has attracted a great interest in recent years (Chu and Lee, 2007; Fischer et al., 2008; Matsushika et al., 2009a). Through this pathway, xylose is reduced to xylitol by NADPH-dependent xylose reductase (XR) encoded by *XYL1* and then xylitol is converted into xylulose, which can be converted by NAD^+^-dependent xylitol dehydrogenase (XDH) encoded by *XYL2*. Xylulose can be phosphorylated into xylulose-5-phosphate (X-5P) by xylulokinase (XK) for metabolism into the non-oxidative pentose phosphate pathway (PPP) and the glycolysis pathway. Another pathway to convert xylose into xylulose in one step is the xylose isomerase pathway (Chu and Lee, 2007; Matsushika et al., 2009a). Functional expression of a xylose isomerase in yeast was achieved after efforts of a few decades (Chu and Lee, 2007). Pronk and co-workers successfully cloned a xylose isomerase from fungi *Pyromyces sp*. (ATCC 76762) (Kuyper et al., 2003). However, the ethanol productivity of the xylose isomerase pathway was 1.6-fold lower than that of XR-XDH pathway although the overall ethanol yield (0.43 g/g) was 30.0% higher (Karhumaa et al., 2007). Recently more research attention was paid on the XR-XDH pathway for improved ethanol yield and productivity. However, the ethanol yield from xylose fermentation is very low due to the fact that a large fraction of consumed xylose is secreted as its reduction product xylitol (Jeppsson et al., 2006; Chu and Lee, 2007; Matsushika et al., 2009a).

Formation of xylitol is mainly ascribed to the difference in co-factor preference between XR and XDH, which causes intracellular redox imbalance and leads to xylitol accumulation (Jeffries, 2006; Chu and Lee, 2007). This hypothesis has been supported by the observation that alteration of cofactor dependence of XR or XDH by directed evolution decreased xylitol production and increased ethanol yield (Jeppsson et al., 2006; Matsushika et al., 2008; Petschacher and Nidetzky, 2008; Bengtsson et al., 2009). Genome-scale modeling of the xylose pathway with NADP^+^-dependent XDH simulated an increased ethanol production by 24.7% and reduced fermentation time by 70% (Kao et al., 2011).

Besides the approach to alter the coenzyme preference of XR or XDH, balance of the XR/XDH activity can also improve xylose utilization (Walfridsson et al., 1997; Karhumaa et al., 2006). Walfridsson and co-workers observed that a strain with an XR/XDH activity ratio of 17.5 produced 0.82 g xylitol/g xylose while a strain having a ratio of 0.06 didn't produce xylitol (Walfridsson et al., 1997). Proper balance of enzymatic reactions of the pathway is required for high productivity to avoid accumulation of toxic intermediates (Dueber et al., 2009; Ajikumar et al., 2010; Bond-Watts et al., 2011). Production of xylitol consumes NADPH and destroys the balance of reductive hydrogen pool (Jeppsson et al., 2002). Therefore, balance of *XYL1* and *XYL2* in xylose conversion pathway can maximize pathway flux, recycle NADPH generation and improve ethanol production. Tuning the promoter strengths or plasmid copy number is a commonly used strategy to balance pathway flux. Lu and Jeffries (2007) shuffled two promoters for key genes *TKL1*, *TAL1*, and *PYK1* in xylose metabolic pathway to optimize the xylose fermentation. The optimal version of *GND2p-TAL1-HXK2p-TKL1-HXK2p-PYK1* was identified by calculation of volumetric ethanol production (Lu and Jeffries, 2007). In this study, we applied the similar strategy to optimize the initial xylose metabolic pathway. Three promoters in *S. cerevisiae* were used to manipulate the expression level of *XYL1* and two plasmids of different gene copy numbers to modulate the expression level of *XYL2* for balance of *XYL1* and *XYL2*.

Chassis is also a factor that should be taken into consideration in improving xylose fermentation (Boghigian et al., 2012). The enzymes in non-oxidative PPP have distinct activities in different chassises, causing varied capacities to metabolize xylulose. Consequently, the different xylulose metabolism resulted in various xylose metabolism styles, as reported that different chassises carrying the identical xylose pathways differed in xylose fermentation (Matsushika et al., 2009b,c; Hector et al., 2011). So far no parameter has been defined to measure the genetic fitness of a chassis for expression of a heterologous xylose pathway. Thus it is plausible that evaluation of different chassises could be part of the avenues to improve xylose fermentation.

In the present study, we reported the construction and optimization of a xylose-utilizing module containing *XYL1* and mutated *XYL2* (D207A/I208R/F209S/N211R) (*mXYL2*) in *S. cerevisiae*. Different parts of the xylose-utilizing module were balanced by fine-tuning the expression levels of *XYL1* and *mXYL2* through various promoters for controlling *XYL1* and different copy numbers for *mXYL2*. Two yeast chassises were selected for functional expression of the xylose-utilizing modules. The results showed that the combined strategy has improved xylose-fermentation to ethanol in *S. cerevisiae*.

## Methods

### Strains and media

Yeast *S. cerevisiae* strain W303a (MATa, leu2-3,112 his3-11, 15 ura3-1 ade2-1 trp1-1 can1-100 rad5-535) and L2612 (MATalpha, leu2-3, leu2-112, ura3-52, trp1-298 can1 cyn1 gal+), a gift from Prof. Thomas Jeffries at University of Wisconsin–Madison, were used as host strains. *E. coli* DH5α was used for common genetic manipulation. *E. coli* DH5α was grown in LB medium (10 g/l tryptone, 5 g/l yeast extract, 10 g/l sodium chloride) supplemented with 100 mg/l ampicillin when used for plasmid construction. Yeast cells were routinely cultured in yeast extract peptone dextrose (YPD) medium (10 g/l yeast extract, 20 g/l peptone, 20 g/l glucose). To select transformants using ura3 or leu2 auxotrophic marker, synthetic component (SC) medium was used, which contained 6.7 g/l YNB, 20 g/l glucose, 20 g/l agar, and 2 g/l amino acid dropout mixture missing uracil or leucine when necessary. Aerobic growth or anaerobic fermentation was performed in YPX medium (10 g/l yeast extract, 20 g/l peptone, 20 g/l xylose).

### Construction of recombinant plasmids

Plasmids and primers used in the study are described in Tables 1 and 2, respectively. Genes *XYL1* and *mXYL2* were codon-optimized and chemically synthesized by Geneart AG (Regensurg, Germany). The *XYL1* sequence contained the optimized ORF sequence of XR from *S. Stipitis* CBS6054 and *PGK1* terminator sequence with *Pst I* at the 5' end and *Hind III*, *Spe I* at the 3′ end, respectively. Similarly, gene *mXYL2* contained the ORF sequence of mutant XDH and *PGK1* terminator sequence. Restriction site *Pst I* was added at the 5′ end and *BamH I*, *Hind III* at the 3′ end. The *XKS1* including its ORF and native terminator, *ADH1* promoter (*ADH1*), truncated *ADH1* promoter (*tADH1*) and *PGK1* promoter were amplified from genomic DNA of strain L2612 and checked by sequencing.

**Table 1:** **Strains and plasmids used in the study**.

**Strains/plasmids**	**Relevant genotype**	**Source or references**
**STRAINS**		
W303a	*MATa,leu2,his3,ura3,ade2,trp1,can1,rad5*	Jeppsson et al., 2003
L2612	*MATalpha,leu2,ura3, trp*1	Jin and Jeffries, 2003
W303tAR	W303a, YIplac211-I	This study
W303AR	W303a, YIplac211-II	This study
W303PR	W303a, YIplac211-III	This study
W303C	W303a, YIplac211	This study
L2612C	L2612, YIplac211	This study
L2612tAR	L2612, YIplac211-I	This study
L2612AR	L2612, YIplac211-II	This study
L2612PR	L2612, YIplac211-III	This study
L2612PR-MD	L2612PR, pRS425-XDH	This study
L2612PR-D	L2612PR, pRS305-XDH	This study
L2612PR-MC	L2612PR, pRS425	This study
L2612PR-C	L2612PR, pRS305	This study
**PLASMIDS**		
pUC18	Gene cloning	Takara
pTA2	Gene cloning	TOYOBO
YIplac211	*URA3*, an integrative plasmid	ATCC87593
pRS305	*LEU2*, an integrative plasmid	Sikorski and Hieter, 1989
pRS425	*LEU2*,a multicopy plasmid	ATCC77106
YIplac211-I	YIplac211,*tADH1p-XYL1-PGK1t*, *PGK1p-mXYL2-PGK1t,PGK1p-XKS1-XKS1t*	This study
YIplac211-II	YIplac211,*ADH1p-XYL1-PGK1t, PGK1p-mXYL2-PGK1t,PGK1p-XKS1-XKS1t*	This study
YIplac211-III	YIplac211,*PGK1p-XYL1-PGK1t, PGKp-mXYL2-PGKt,PGKp-XKS1-XKS1t*	This study
pRS305-XDH	pRS305,*PGK1p-mXYL2-PGK1t*	This study
pRS425-XDH	pRS425, *PGK1p-mXYL2-PGK1t*	This study

**Table 2:** **Primers used in the study**.

**Primer name**	**Sequences**
tADH1pF	gggAAGCTTACACTGCCTCATTGATGGTG
ADH1pF	gggAAGCTTAAGAAATGATGGTAAATGAAATA
ADH1pR	gggCTGCAGTGTATATGAGATAGTTGATT
PGK1pF1	gggAAGCTTGATTCCTGACTTCAACTCAAGACG
PGK1pR1	gggCTGCAG TGTTTTATATTTGTTGTAAA
PGK1pF2	gggGGATCCGATTCCTGACTTCAACTCAAGACG
PGK1pR2	gggCTGCAG TGTTTTATATTTGTTGTAAA
PGK1pF3	gggGAATTCGATTCCTGACTTCAACTCAAGACG
PGK1pR3	gggGGATCCTGTTTTATATTTGTTGTAAA
XKS1F	gggGGATCCATGTTGTGTTCAGTAATTCAGAGACAG
XKS1R	gggCTGCAGGAATTCGAGCTCGAGATGATTTAACAATAAC
M13R	TGTAAAACGACGGCCAGTG
Xyl1R	GAGCAAATTCGATCAATCTAGGT
Xyl2F	GCTCCAGGTGGTAGATTTGTC
Xks1F	CGGATGCCTGTGGTATGAA
M13F	CAGGAAACAGCTATGACC

*Underlined sequences are restriction enzyme recognition sites*.

Plasmids YIplac211-I, YIplac211-II, and YIplac211-III were constructed as follows. First, the three promoters were cloned into vector pTA2 using primers described in Table 2. The 1.26 kb *Pst I*-*Spe I* fragment of gene *XYL1* was inserted into pTA2 to form three types of *XYL1* expression cassettes. The cassettes were released by *Hind III* digestion and inserted into *Hind III* site in plasmid YIplac211 resulting in plasmids YIplac211-XR (*tADH1*), YIplac211-XR (*ADH1*), YIplac211-XR (*PGK1*). The orientations of cassettes were checked by PCR using primers M13R and Xyl1R.

On the basis of serial YIplac211-XR plasmids, *mXYL2*, and *XKS1* expression cassettes were inserted sequentially. The *mXYL2* expression cassette was constructed in pUC18 as follows. The *PGK1* promoter was amplified from genomic DNA using primers *PGK1*pF2 and *PGK1*pR2 and inserted into *BamH I* and *Pst I* sites in pUC18. Next, the DNA fragment of *mXYL2* was inserted into *Pst I* and *Hind III*. After that, the *mXYL2* cassette sequence was released by *BamH I* and cloned into *BamH I* site in plasmids YIplac211-XR (*tADH1*), YIplac211-XR (*ADH1*), and YIplac211-XR (*PGK1*). The correct clones were verified by enzymatic digestion and PCR test using primers Xy12F and M13F, generating plasmids YIplac211-XR (*tADH1*) XDH, YIplac211-XR (*ADH1*) XDH and YIplac211-XR (*PGK1*) XDH.

**Figure 1:**
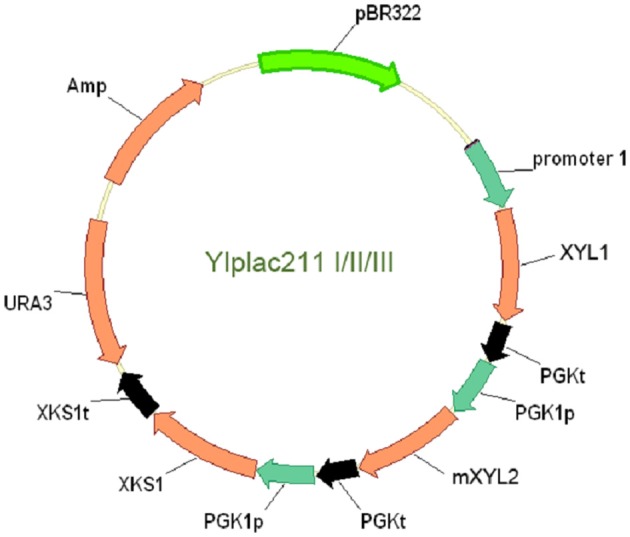
**The physical map of the plasmids YIplac211-I, II, and III for co-expression of *XYL1*, *mXYL2* and *XKS1* in yeast W303a and L2612**. Promoter 1 represents *tADH1*, *ADH1*, and *PGK1* promoters in YIplac211-I, II and III. The transcription orientations of the three genes were the same in the plasmids.

To clone *XKS1* expression cassette into the above YIplac211-XRXDH plasmids, *XKS1* sequence with restriction sites was amplified from genomic DNA and cloned into *BamH I* and *Pst I* sites of pUC18, yielding plasmid pUC18-XKS1. The *PGK1* promoter sequence amplified from genomic DNA using primers *PGK1*pF3 and *PGK1*pR3 was cloned into *EcoR I* and *BamH I* sites in plasmid pUC18-XKS1 to form the *XKS1* expression cassette. The cassette was then subcloned into *EcoR I* site in analogue YIplac211-XRXDH plasmids. The clones with the correct orientation were checked by enzymatic digestion and PCR test using primers XKS1F and M13F. The resultant plasmids were designated as YIplac211-I, YIplac211-II, YIplac211-III (Figure 1).

PRS305-XDH and pRS425-XDH were constructed by inserting *mXYL2* cassette mentioned above into *BamH I* site of pRS305 (Sikorski and Hieter, 1989) and pRS425, respectively.

### Yeast transformation

Yeast transformation was carried out by the lithium acetate method (Gietz et al., 1995). Plasmids of YIplac211, YIplac211-I, YIplac211-II, and YIplac211-III were linearized by *ApaI* before transformation. Plasmids pRS305 and pRS305-XDH were linearized by *AflII* prior to integration into yeast genome. Transformants were selected on SC medium containing 20 g/l glucose. Amino acids and nucleotides were added when necessary.

### Fermentation

For inocula preparation, cells were cultivated in YPD medium or SC medium to maintain the plasmids when necessary at 30°C under aerobic conditions. Cells at mid-exponential phase were harvested by centrifugation at 1432×g for 5 min. For aerobic growth, cells were grown in 50 ml YPX medium in 250 ml shake-flasks with an initial optimal density at 600 nm (OD_600_) of 0.5 (Model 722 grating spectrometer, Shanghai No. 3 Analysis Equipment Factory, Shanghai, China). For anaerobic fermentation, harvested cells were inoculated to an OD_600_ of 2.0 in 100 ml YPX medium in 250 ml shake-flasks sealed by a rubber stopper with a needle to release CO_2_ produced during the fermentation process. Both fermentations were conducted at 30°C at 150 rpm unless noted specifically. All the experiments were repeated independently.

### Measurement of cell growth and analysis of fermentation products

Cell density was monitored by measuring the absorbance of the culture at 600 nm with a spectrometer (Model 722 grating spectrometer, Shanghai No. 3 Analysis Equipment Factory, Shanghai, China). Samples were taken periodically from cultures and centrifuged at 9600×g for 5 min. Supernatant was collected for analysis on a HPLC system consisting of a HPLC pump (Waters 1515), a Bio-Rad HPX-87H column (Bio-Rad, Hercules, CA) and a refractive index detector (Waters 2414) (Ding et al., 2012). The column was eluted at 65°C with 5 mM sulfuric acid at a flow rate of 0.6 ml/min.

## Results

### Effects of different levels of XYL1 on aerobic xylose consumption

**Figure 2:**
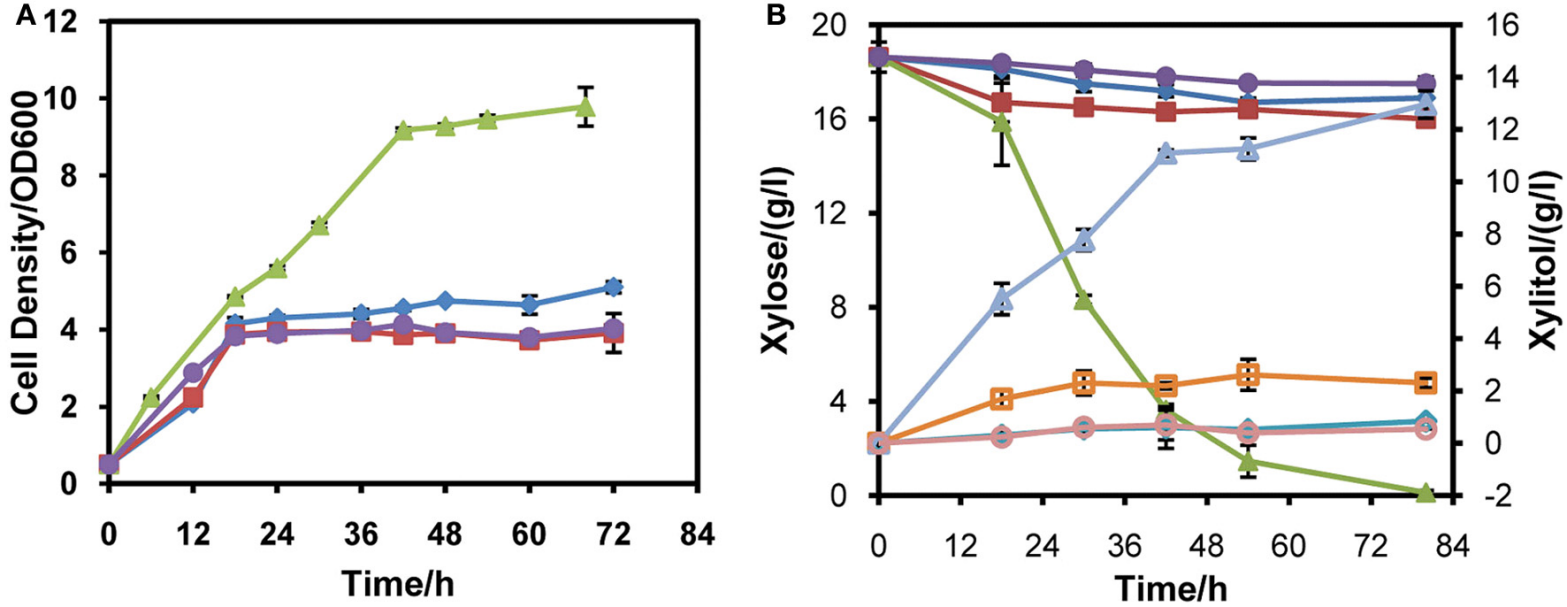
**Aerobic xylose consumption of strains W303tAR, W303AR, W303PR, and W303C in YPX medium**. **(A)** growth profiles, **(B)** xylose consumption and xylitol production. Three promoters were applied to express *XYL1* for balance of *XYL1* and *XYL2*. W303tAR, W303AR, and W303PR harbored truncated *ADH1* promoter, intact *ADH1* promoter and *PGK1* promoter, respectively. W303C was the control strain. The aerobic cell growth was conducted in 50 ml YPX medium containing 20 g/l xylose. Symbols: diamonds, W303tAR; squares, W303AR; triangles, W303PR; circles, W303C. In panel **(B)**, closed symbols represent xylose concentration and open symbols represent xylitol concentration. The concentration of glycerol is not presented (see Table 3). Ethanol was not detected in the media. The results shown were the mean value of duplicate experiments.

To probe the appropriate expression level of *XYL1* for matching *mXYL2*, three promoters were applied to express *XYL1* along with *PGK1* promoter expressing *mXYL2*. Two versions of *ADH1* promoters, full-length *ADH1* (*ADH1*) and truncated *ADH1* (*tADH1*), were tested here (Liu and Hu, 2010). The constructed xylose conversion pathways were expressed in chassis W303a, resulting in strains W303tAR, W303AR, and W303PR of those the *XYL1* was expressed by the promoters *tADH1*, *ADH1*, and *PGK1*, respectively. Aerobic growth was conducted to characterize these strains compared with the control strain W303C (Figure 2). Strains W303tAR, W303AR, and W303PR consumed 1.92, 2.71 and 17.42 g/l xylose, respectively, corresponding to 74.5%, 146.3 %, and 14.8-fold increase than strain W303C which consumed 1.10 g/l xylose (Table 3). The xylitol yield in W303tAR and W303C was nearly the same, whereas the xylitol yield of W303AR and W303PR was 73.5% and 30.6% higher than that of W303C. The glycerol yield in W303tAR, W303AR, and W303PR was lower than that of the control strain W303C. The biomass yield from W303tAR and W303PR were 3.86, and 2.58 folds of that in W303C. Although strain W303AR consumed more xylose than W303tAR and W303C, nearly no biomass was produced because most of the assimilated xylose was secreted as xylitol during the growing process, indicating severe imbalance of the xylose metabolic pathway with *XYL1* promoted by *ADH1* (Table 3). In summary, only promoter *PGK1* facilitated xylose uptake for strain W303a, while the other promoters failed.

**Table 3:** **Xylose consumption of *S. cerevisiae* strains W303tAR, W303AR, W303PR, and W303C under aerobic conditions**.

**Strain**	**Consumed xylose (g/l)^a^**	**Yield (g/g consumed xylose)^a^**		
		**Xylitol**	**Glycerol**	**Biomass**
W303TAR	1.92 ± 0.18	0.44 ± 0.03	0.046 ± 0.004	0.27 ± 0.09
W303AR	2.71 ± 0.52	0.85 ± 0.05	0.018 ± 0.002	0.18 ± 0.01
W303PR	17.42 ± 1.02	0.64 ± 0.01	0.006 ± 0.000	0.01 ± 0.00
W303C	1.10 ± 0.05	0.49 ± 0.01	0.068 ± 0.005	0.07 ± 0.01

*Strains W303tAR, W303AR, W303PR were integrated with xylose conversion pathway with XYL1 controlled by truncated ADH1 promoter, intact ADH1 promoter and PGK1 promoter, respectively. W303C was the control strain. The growth was conducted in 50 ml YPX medium containing 20 g/l xylose under aerobic conditions. The initial cell density was adjusted to 0.5 (OD_600_)*.

a *The results were calculated based on metabolites concentration measured at 80 h of the fermentation*.

To investigate the capacity to produce ethanol from xylose, xylose fermentation by strain W303PR was conducted with xylose concentration up to 50 g/l at different aeration rate ranging from 0 to 0.556 vvm (volumes of air per volume of liquid per min) in a fermentor (Baoxin Biotech Ltd., Shanghai, shanghai, China). However, ethanol was not produced while a large amount of xylitol was formed. The reason for this may be that metabolic flux was channeled into TCA cycle induced by xylose (Jin et al., 2004), consistent with cell growth in xylose fermentation (Figure 2A). These results indicate that W303a is not an appropriate chassis for xylose pathway expression, which leads us to chassis yeast L2612.

### Comparison of two chassis for expressing XR/XDH pathway

**Figure 3:**
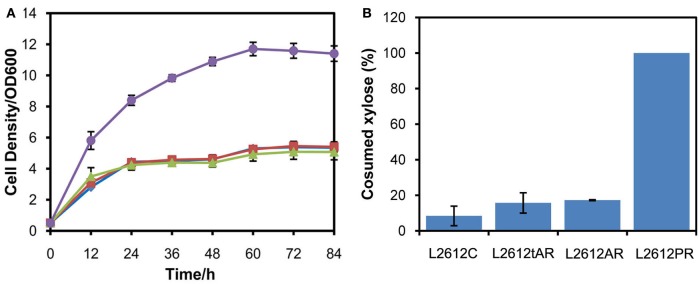
**Comparison of growth profiles (A) and xylose uptake capacities (B) of three recombinant strains derived from yeast strain L2612 and control strain L2612C in YPX medium under aerobic conditions**. Symbols: squares, L2612C; diamonds, L2612tAR; triangles, L2612AR; circles, L2612PR. Strains L2612tAR, L2612AR, L2612PR were integrated with xylose conversion pathway with *XYL1* controlled by truncated *ADH1* promoter, full-length *ADH1* promoter and *PGK1* promoter in yeast L2612. L2612C was the control strain. Xylose uptake capacities are presented as the percentage of consumed xylose after 72 h. The results shown were the mean value of duplicate experiments.

Another yeast chassis L2612 was examined to express the three xylose pathways and compared with chassis W303a. Similar to the results obtained in W303a, the xylose pathways in which *XYL1* was under the control of *ADH1* or *tADH1* did not work in strain L2612 (Figure 3A). Only the strain L2612PR harboring the strongest promoter *PGK1* to express *XYL1* grew rapidly under aerobic conditions. Consistently, L2612tAR, L2612AR, and parent strain L2612C consumed 15.7, 17.3, and 8.4% of the total xylose, respectively (Figure 3B). In contrast strain L2612PR consumed all the xylose.

The performances of L2612PR and W303PR were compared under conditions of different oxygen supply. As shown in Table 4, L2612PR produced less byproduct xylitol than W303PR under different oxygen supply, indicating XR and XDH are more balanced in L2612 than W303PR. Additionally, the glycerol yield in L2612PR was approximately half of that in W303PR under aerobic conditions. Accompanying decreased oxygen supply, the xylose consumption rates in W303PR and L2612PR decreased dramatically by 20.3% and 44.8%, respectively, indicating the xylose metabolism of L2612PR is more dependent on oxygen (Table 4). A previous microarray study in the xylose-fermenting strain YSX3 derived from L2612 also supports the xylose metabolism style that xylose metabolic flux is prone to respiratory pathway. Under oxygen-limited conditions, respiratory pathway is blocked leading to decreased xylose metabolism rate (Jin et al., 2004).

**Table 4:** **Comparison of xylose consumption performances of W303PR and L2612PR under conditions of different oxygen supply**.

	**W303PR**		**L2612PR**	
	**Oxygen-limited (100 rpm)**	**Aerobic (150 rpm)**	**Oxygen-limited (100 rpm)**	**Aerobic (150 rpm)**
r^a^_xylose_	0.247 ± 0.00	0.310 ± 0.03	0.170 ± 0.03	0.308 ± 0.01
Y^b^_xylitol_	0.59 ± 0.03	0.60 ± 0.03	0.47 ± 0.02	0.27 ± 0.02
Y^c^_glycerol_	0.08 ± 0.01	0.07 ± 0.01	0.09 ± 0.01	0.04 ± 0.01

*W303PR and L2612PR had the same xylose pathway PGK1p-XYL1-PGKp-mXYL2-PGKp-XKS1 except the host strains. The host strains of W303PR and L212PR are W303a and L2612, respectively. The different oxygen supply was obtained by changing the shaking speed. The initial cell density was adjusted to 0.5 (OD_600_). The results are the average of two independent experiments after 72 h fermentation*.

a *Volumetric xylose consumption rate is given in g/l/h*.

b *Xylitol yield is given in g/g consumed xylose*.

c *Glycerol yield is given in g/g consumed xylose*.

Considering byproducts xylitol and glycerol production, it is concluded that yeast L2612 is a better chassis to express xylose pathway for ethanol production. L2612PR could be a starting strain for further modification to decrease xylitol production and increase metabolic flux to ethanol under anaerobic conditions.

### Effects of different levels of *XYL2* on xylose fermentation

Because XDH is directly associated with the conversion of xylitol to xylulose which can be metabolized by the non-oxidative PPP, we postulated that enhancement in *mXYL2* expression could reduce xylitol secretion, increase the carbon flux to the non-oxidative PPP and consequently increase ethanol production. To verify the postulation, we introduced different copies of *mXYL2* into strain L2612PR. In addition, a parallel investigation of the effects of different expression levels of *mXYL2* on xylose fermentation was also conducted.

**Figure 4:**
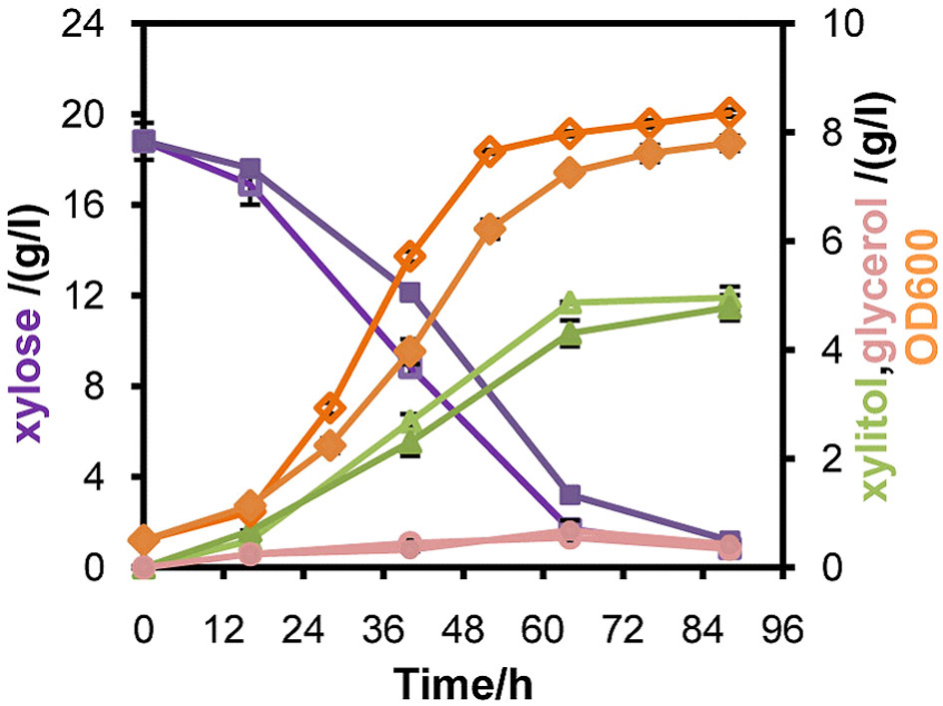
**Comparison of the performances of recombinant strain L2612PR-D (Open symbols) and its control strain L2612PR-C (Closed symbols) in xylose under aerobic conditions**. L2612PR-D was integrated with one additional copy of *mXYL2* in the genome. The aerobic growth was carried out in 50 ml YPX medium with 20 g/l xylose. The initial OD_600_ of the culture was 0.5. Symbols: diamonds, OD_600_; squares, xylose consumption; triangles, xylitol concentration; circles, glycerol concentration. The results shown were the average value of two independent experiments.

We first compared the performances of the constructed strains under aerobic conditions. L2612PR-MD harboring multiple copies of *mXYL2* showed small differences in growth and product distribution compared with the control strain L2612PR-MC (Table 5). Different from multiple copies of *mXYL2*, overexpression of *mXYL2* by genomic integration had a significant effect on xylose consumption. Compared with the control strain L2612PR-C, strain L2612PR-D assimilated xylose faster but not significantly (*P* = 0.058) (Figure 4). The average xylose consumption rate of L2612PR-D was 10% higher of that in L2612PR-C (Table 5). However, the xylitol yield, glycerol yield, and biomass yield in L2612PR-D stayed nearly the same as that in L2612PR-C (Table 5).

**Table 5:** **Aerobic xylose consumption of L2612PR-MD, L2612PR-MC, L2612PR-D, and L2612PR-C**.

**Strain**	**r_xylose_^a^**	**Xylitol (g/l)^b^**	**Glycerol (g/l)^b^**	**Yield (g/g consumed xylose)^b^**		
				**Xylitol**	**Glycerol**	**Biomass**
L2612PR-MD	0.240 ± 0.002	4.84 ± 0.20	0.42 ± 0.03	0.27 ± 0.01	0.02 ± 0.00	0.22 ± 0.00
L2612PR-MC	0.257 ± 0.002	5.16 ± 0.04	0.45 ± 0.01	0.29 ± 0.00	0.03 ± 0.00	0.22 ± 0.00
L2612PR-D	0.268 ± 0.001	4.96 ± 0.20	0.34 ± 0.02	0.28 ± 0.01	0.02 ± 0.00	0.22 ± 0.00
L2612PR-C	0.244 ± 0.001	4.78 ± 0.23	0.42 ± 0.02	0.27 ± 0.01	0.02 ± 0.00	0.21 ± 0.00

*mXYL2 are overexpressed in strains L2612PR-MD and L2612PR-D in the form of a multicopy plasmid and genomic integration, respectively. L2612PR-MC and L2612PR-C were the corresponding control strain. The experiment was conducted in 50 ml YPX medium containing 20 g/l xylose under aerobic conditions. The initial cell density was adjusted to 0.5 (OD_600_)*.

a *r_xylose_ the volumetric xylose uptake rate (g xylose/l/h) over 64 h*.

b *The values were calculated after 88 h of fermentation*.

**Figure 5:**
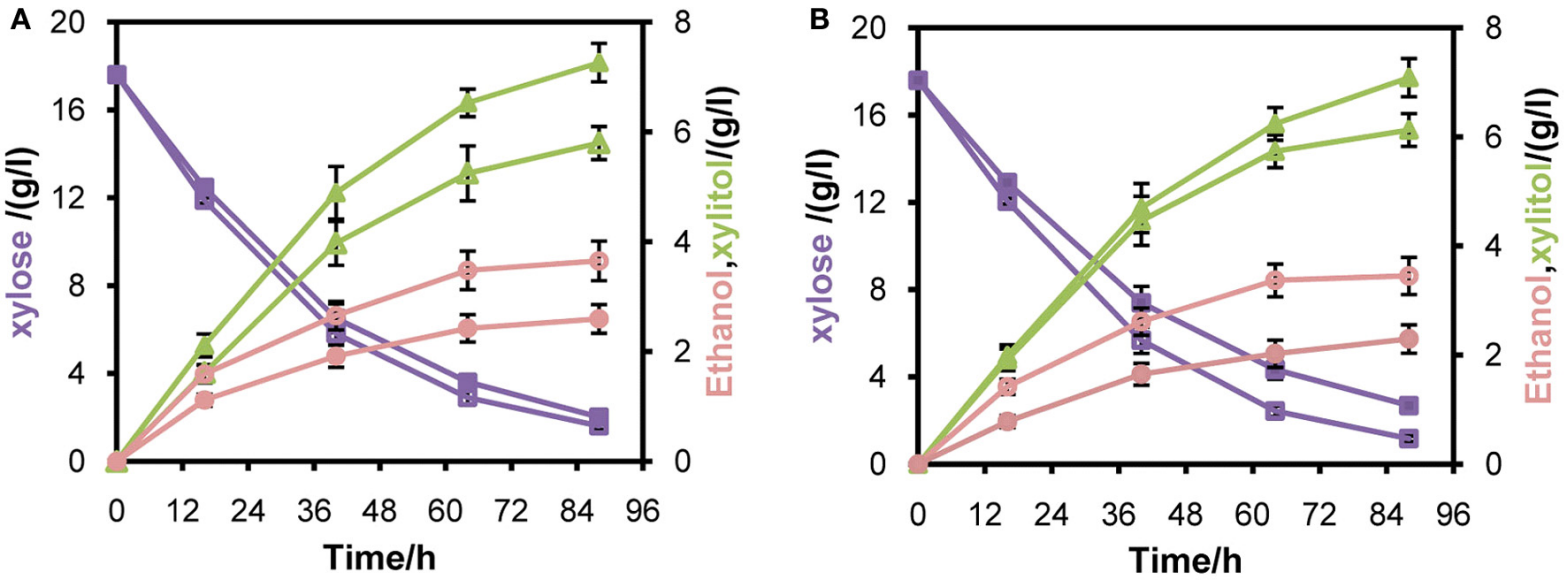
**Anaerobic batch fermentation of L2612PR-MD (open symbols), L2612PR-MC (closed symbols) (A), L2612PR-D (open symbols) and L2612PR-C (closed symbols) (B) in YPX medium**. L2612PR-MD, L2612PR-D had a multicopy plasmid and an integrative plasmid with *mXYL2* based on L2612PR, respectively. L2612PR-MC and L2612PR-C were the corresponding control strains. Figure legends: squares, xylose consumption; triangles, xylitol concentration; circles, ethanol concentration. The concentration of glycerol is not presented (see Table 6). The results shown were the average value of two independent experiments.

To verify whether enhanced *mXYL2* expression had any positive effects on xylose fermentation under anaerobic conditions, the performances of the four strains were compared. As shown in Figure 5, excessive overexpression of *mXYL2* improved xylose uptake in L2612PR-MD and L2612PR-D compared with their control strains. The overall xylose consumption rate and maximal specific xylose uptake rate improved a bit for L2612PR-MD (Table 6). Additionally, L2612PR-MD produced 5.80 g/l xylitol, much less than L2612PR-MC (7.26 g/l) at the end of fermentation (Figure 5A, Table 6). The xylitol yield decreased by 21.7% from 0.46 g xylitol g consumed xylose^−1^ in L2612PR-MC to 0.36 g xylitol g consumed xylose^−1^ in L2612PR-MD. Consistent with reduced xylitol, ethanol production elevated from 2.60 g/l in L2612PR-MC to 3.65 g/l in L2612PR-MD (Figure 5A, Table 6), which was a 35.2% increase. Similarly, the xylitol yield reduced by 21.3% from 0.48 g xylitol g consumed xylose^−1^ in L2612PR-C to 0.37 g xylitol g consumed xylose^−1^ in L2612PR-D (Table 6, Figure 5B). L2612PR-D produced 50.0% more ethanol than L2612PR-C. Correspondingly, the ethanol yield increased from 0.15 g ethanol g consumed xylose^−1^ in L2612PR-C to 0.21 g ethanol g consumed xylose^−1^ in L2612PR-D, elevated by 40.0%. The average xylose consumption rate increased by 13.0% in L2612PR-D than L2612PR-C. Glycerol yield and biomass yield was the same for L2612PR-D and L2612PR-C (Table 6). However, the glycerol doubled in L2612PR-MD from L2612PR-MC, which may reflect increased flux towards glyceraldehyde 3-phosphate from non-oxidative PPP. Taken together, additional overexpression of *mXYL2* in L2612PR increased the ethanol yield and reduced the xylitol production from xylose, indicating its importance in regulating xylose metabolism by XR/XDH pathway.

**Table 6:** **Summary of anaerobic batch fermentation of L2612PR-MD, L2612PR-MC, L2612PR-D, and L2612PR-C in xylose medium**.

**Strain**	**r_xylose_^a^**	**Ethanol (g/l)^b^**	**Xylitol (g/l)^b^**	**Glycerol (g/l)^b^**	**Yield (g product/consumed xylose)^b^**			
					**Ethanol**	**Xylitol**	**Glycerol**	**Biomass**
L2612PR-MD	0.230 ± 0.001	3.65 ± 0.25	5.80 ± 0.40	0.23 ± 0.04	0.23 ± 0.01	0.36 ± 0.02	0.02 ± 0.00	0.06 ± 0.00
L2612PR-MC	0.221 ± 0.001	2.60 ± 0.54	7.26 ± 0.34	0.14 ± 0.01	0.17 ± 0.01	0.46 ± 0.02	0.01 ± 0.00	0.07 ± 0.01
L2612PR-D	0.234 ± 0.002	3.45 ± 0.14	6.13 ± 0.26	0.19 ± 0.01	0.21 ± 0.01	0.37 ± 0.02	0.01 ± 0.00	0.07 ± 0.01
L2612PR-C	0.207 ± 0.001	2.30 ± 0.16	7.09 ± 0.38	0.15 ± 0.02	0.15 ± 0.01	0.48 ± 0.02	0.01 ± 0.00	0.06 ± 0.01

*The fermentation was carried out in 100 ml YPX medium with 20 g/l xylose under anaerobic conditions. The beginning cell density was OD_600_ 1.0 for all strains. The fermentation continued for 88 h*.

a *r_xylose_ the volumetric xylose consumption rate (g xylose/l/h) over 64 h*.

b *Ethanol, xylitol and glycerol concentrations were determined at 88 h of fermentation*.

## Discussion

In the present study, we applied the combined strategy of pathway balance and chassis optimization for improved xylose fermentation by XR/XDH pathway in *S. cerevisiae*. This approach provided a practical way to optimize xylose metabolic pathway for ethanol production and could be applied in other chemical production schemes.

**Figure 6:**
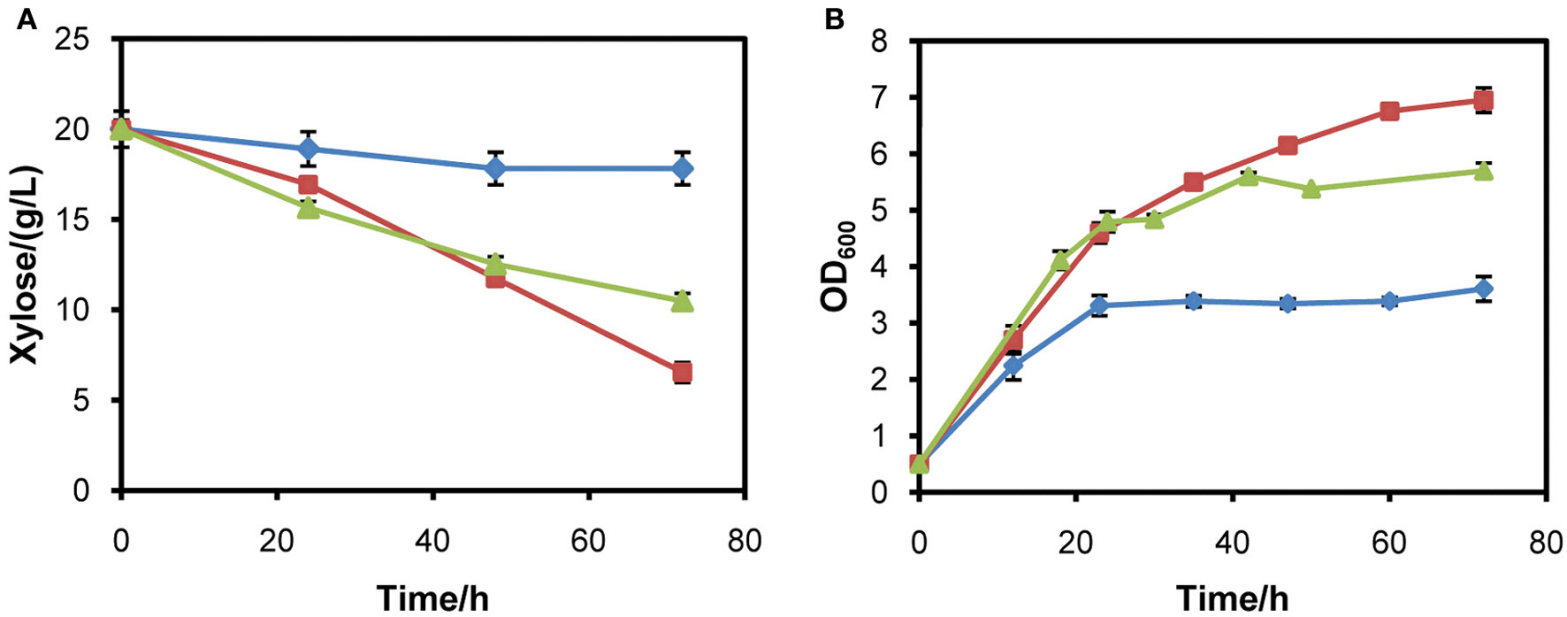
**Aerobic growth of strains W303a-C, W303a-I, and W303a-II in YPX medium. (A)** Xylose consumption. **(B)** Growth profiles. Symbols: diamonds, W303a-C; squares, W303a-I; triangles, W303a-II. W303a-C is the W303a strain harboring a multicopy plasmid of pRS426. In contrast, W303a-I and W303a-II are strains harboring multiple copies of *tADH1p-XYL1-PGK1t* and *ADH1p-XYL1-PGK1t* coupling *XYL2* and *XKS1* on the backbone of plasmid pRS426, respectively. The initial cell density was adjusted to 0.5 (OD_600_).

In optimizing the expression of *XYL1*, it was observed that strains with *XYL1* under the control of *tADH1* or *ADH1* promoter had almost no ability to utilize xylose. Since XR determines the entry of xylose into the xylose pathway in the first step, the poor abilities to assimilate xylose may be ascribed to insufficient activity of XR. After increasing activity of XR by using *PGK1* promoter, the strain was able to assimilate and metabolize xylose faster. The result demonstrates that high activity of XR is necessary for rapid xylose metabolism and ethanol fermentation. In a previous study, an additional copy of XR increased xylose consumption rate by 1.7 fold and resulted in a 55% lower xylitol yield (Jeppsson et al., 2003). It was also observed that strain INVSc1 with *XYL1* controlled by the promoter *ADH1* consumed 9% less xylose than the strain harboring the promoter *PGK1* (Matsushika and Sawayama, 2008). The results reported in the literature and our experiments further verify the conclusion that XR activity largely determines the rate of xylose consumption and has important effects on products distribution. Additionally, in our study it was observed that the constitutive strong promoter *ADH1* was almost unable to initiate xylose metabolism. This may be ascribed to the requirement of a much higher expression of *XYL1* in our strains than strains used elsewhere. Moreover, we placed the same pathway in a multicopy plasmid and the resulting recombinant strains obtained the ability to utilize xylose and grow in xylose medium (Figure 6). It further indicates that *ADH1* promoter is not able to facilitate enough XR activity for rapid xylose metabolism in W303a. Employment of stronger promoters such as *TDH3* for *XYL1* expression might further enhance xylose consumption rate and shorten the fermentation time.

Besides high expression of *XYL1*, high expression of *XYL2* is also necessary to decrease xylitol production. Overexpression of *mXYL2* by a multicopy plasmid or genomic integration in strain L2612PR optimized the xylose metabolic pathway for improved ethanol production. These results strongly support the conclusion that high activity of XDH is required for efficient xylose metabolism, in agreement with previous research (Jin and Jeffries, 2003; Karhumaa et al., 2006; Krahulec et al., 2009; Kim et al., 2012). Higher XDH activity may increase the flux from xylitol to xylulose and thereafter flux to central metabolism through phosphorylation and non-oxidative PPP (Karhumaa et al., 2006). In Krahulec and co-workers' research, the xylose pathway with mutant XDH with three-fold higher specificity on NADP^+^ than NAD^+^ did not improve ethanol production or decrease xylitol accumulation (Krahulec et al., 2009). The authors suggested the failure resulted from low activity of XDH since cofactor balance had been attempted. Jin's work also confirmed the importance of XDH by observing that overexpression of XDH did reduce xylitol secretion (Jin and Jeffries, 2003). The 11.25-fold increase in XDH activity caused a 50% reduction in xylitol formation by using a multicopy of *XYL2* instead of genomic integration of *XYL2*. Different from his work without overexpression of *XKS1*, we also over-expressed *XKS1* to enhance XK activity and significant increase in ethanol production was obtained. In his work, as much as 3 g/l xylulose was produced during xylose fermentation and the highest ethanol yield reached about 0.04 g/g, much lower than that in L2612PR-D and L2612PR-MD which originated from the same yeast chassis L2612, implying that the low XK activity limited the conversion of xylulose to non-oxdative PPP and ethanol production. This result indicates that sufficient XK activity contributes to efficient ethanol production from xylose.

Moreover, overexpression of *XYL2* by a multicopy plasmid did not reduce xylitol secretion and biomass formation in aerobic conditions. However, overexpression of *XYL2* by integration of one additional copy accelerated xylose uptake rate despite of no improvement in xylitol formation. Adverse effect of grave overexpression of *XYL2* may be responsible for this. A previous study supports the hypothesis that introduction of a multicopy plasmid pRS424-PGK1p-XYL2 in the xylose-fermenting yeast YSX3 slows down xylose uptake while genomic integration of PGK1p-XYL2 accelerates xylose uptake rate by 36.5% (Kim et al., 2012). “Just enough” overexpression of *XYL2* is essential for efficient xylose fermentation. Further optimization of *XYL2* by probing promoters of different strengths possibly achieves a better xylose-fermenting strain in our case.

As for the high xylitol yield in L2612PR-D, this may be ascribed to the limited flux of the non-oxidative pathway which compelled increased carbon flux to channel into xylitol. In a previous study, upregulation of PPP genes increased the specific growth rate by 2-fold (Karhumaa et al., 2005). Under aerobic conditions, the carbon flux was enlarged compared to anaerobic conditions, which has been observed in other studies (Jouhten et al., 2008). The limited activities of non-oxidative PPP enzymes restrict the flux of carbon source toward glycolytic pathway and excessive carbon source leaks out in the form of xylitol. A metabolic flux analysis and proteomic assay of key enzymes in PPP and glycolitic pathway could verify the hypothesis and provide new clues for further engineering.

Chassis optimization for expression of XR/XDH pathway is another approach for efficient xylose fermentation besides direct regulation of heterogeneous metabolic pathway. The performances of different yeast strains having identical xylose pathways vary in xylose fermentation. Matsushika and co-workers compared the xylulose fermentation of nine industrial diploid strains and the best chassis IR-2 consumed xylulose faster than other candidate strains (Matsushika et al., 2009c). The expression of XR/XDH pathway in IR-2 allowed an efficient xylose-fermenting strain MA-R5. The comparison indicated that the downstream pathway or xylulose transport system for xylulose metabolism differed largely in these strains. Another example of the inconsistency is that strain TMB3066 grows slower than one of the best xylose-fermenting strain RWB217, both of which have identical xylose isomerase pathways and originate from CEN.PK laboratory strains (Karhumaa et al., 2007). The authors claimed that the differences may result from higher activities of XK or the PPP enzymes in RWB217. Likely, differences in enzymatic activities of the related pathway such as PPP and xylose transportation system, and different regulatory modification over metabolic pathway may contribute to the distinct behavior of W303PR and L2612PR. Systematic comparison between W303PR and L2612PR can give rise to new target genes or networks for improving xylose fermentation further (Wahlbom et al., 2003; Karhumaa et al., 2009).

In optimizing the expression level of *XYL1*, we used different promoters with varied strengths. However, this process was also complemented by applying a promoter library composed of promoter mutants. The mutants with various strengths were assembled into a genetic network and the desired function was obtained by sampling the continuum of gene expression at a series of discrete points or by model-guided rational design (Alper et al., 2005; Hammer et al., 2006; Ellis et al., 2009; Du et al., 2012). The use of such a promoter library made the optimization faster and easier due to the sequence identity or similarity of promoter mutants and thus the construction of genetic network could be accomplished by common cloning manipulation or DNA assembler method (Shao et al., 2009). Furthermore, the introduction of promoters stemming from the same source would avoid unpredicted regulation disorders caused by using promoters of different sources. In future work, such a promoter library can be applied for optimizing the xylose metabolic pathway with additional efforts.

## Conclusion

A xylose pathway composed of *XYL1*, m*XYL2*, and *XKS1* was constructed. Three promoters *tADH1, ADH1*, and *PGK1* were used to modulate the relative expression levels of *XYL1* and m*XYL2*. The results showed that only the strongest promoter *PGK1* facilitated xylose uptake and metabolism in the constructed strains, demonstrating that it is necessary for the high activity of XR in xylose fermentation. Comparison of the fermentation performances between the constructed strains from chassis W303a and L2612 led to a more efficient xylose-fermenting strain L2612PR, which derived from strain L2612. To enhance the expression of m*XYL2*, an extra copy or multiple copies of *mXYL2* was introduced, leading to the generation of strains L2612PR-MD and L2612PR-D, which exhibited 21% lower xylitol production and 35–40% higher ethanol production. The results indicated the importance of XDH in reducing xylitol accumulation and maximizing the flux to downstream xylose metabolic pathway. In summary, our results have demonstrated that it is effective to combine chassis optimization and heterogeneous pathway balance in constructing ethanolic xylose-fermenting *S. cerevisiae*.

### Conflict of interest statement

The authors declare that the research was conducted in the absence of any commercial or financial relationships that could be construed as a potential conflict of interest.
